# Critical developmental windows for morphology and hematology revealed by intermittent and continuous hypoxic incubation in embryos of quail (*Coturnix coturnix*)

**DOI:** 10.1371/journal.pone.0183649

**Published:** 2017-09-19

**Authors:** Warren W. Burggren, Nourhan A. Elmonoufy

**Affiliations:** Department of Biological Sciences, University of North Texas, Denton, TX, United States of America; Laboratoire de Biologie du Développement de Villefranche-sur-Mer, FRANCE

## Abstract

Hypoxia during embryonic growth in embryos is frequently a powerful determinant of development, but at least in avian embryos the effects appear to show considerable intra- and inter-specific variation. We hypothesized that some of this variation may arise from different protocols that may or may not result in exposure during the embryo’s critical window for hypoxic effects. To test this hypothesis, quail embryos (*Coturnix coturnix)* in the intact egg were exposed to hypoxia (~15% O_2_) during “early” (Day 0 through Day 5, abbreviated as D0-D5), “middle” (D6-D10) or “late” (D11-D15) incubation or for their entire 16–18 day incubation (“continuous hypoxia”) to determine critical windows for viability and growth. Viability, body mass, beak and toe length, heart mass, and hematology (hematocrit and hemoglobin concentration) were measured on D5, D10, D15 and at hatching typically between D16 and D18 Viability rate was ~50–70% immediately following the exposure period in the early, middle and late hypoxic groups, but viability improved in the early and late groups once normoxia was restored. Middle hypoxia groups showed continuing low viability, suggesting a critical period from D6-D10 for embryo viability. The continuous hypoxia group experienced viability reaching <10% after D15. Hypoxia, especially during late and continuous hypoxia, also inhibited growth of body, beak and toe when measured at D15. Full recovery to normal body mass upon hatching occurred in all other groups except for continuous hypoxia. Contrary to previous avian studies, heart mass, hematocrit and hemoglobin concentration were not altered by any hypoxic incubation pattern. Although hypoxia can inhibit embryo viability and organ growth during most incubation periods, the greatest effects result from continuous or middle incubation hypoxic exposure. Hypoxic inhibition of growth can subsequently be “repaired” by catch-up growth if a final period of normoxic development is available. Collectively, these data indicate a critical developmental window for hypoxia susceptibility during the mid-embryonic period of development.

## Introduction

Normal morphological and physiological development of the avian embryo, as well as successful hatching, depends on appropriate temperature as well as ambient partial pressures of oxygen, carbon dioxide, and water vapor–for an entry into the voluminous, long-standing literature see [1,2]. Hypoxia is a known stressor affecting normal ontogeny. Embryonic responses–both adaptive and maladaptive—to hypoxic incubation in avian embryos include (but are not limited to) whole body growth retardation, reduction in oxygen consumption, specific growth retardation of organs like the beak and toes, heart hypertrophy, changes in heart conduction and cardiac rhythms, pericardial and pulmonary edema, accelerated angiogenesis, stimulated hematopoesis, hemoglobin modifications (including changes in the time course of adult Hb appearance) and red blood cell ATP concentration changes [3–28].

Most of these studies on the effect of hypoxia on avian development have exposed embryos to chronic hypoxia throughout the entire incubation prior to measurements or during the last half of incubation—see [6] for discussion of the most common hypoxic exposure protocols. Far fewer studies have used selective, briefer periods (“pulses”) of hypoxic exposure to probe hypoxic effects during incubation. Moreover, there is little consistency from study to study in duration, intensity or timing of the hypoxic period. All developing animals have so-called “critical windows” for development [29–33]. Studies of critical windows in chicken embryos have investigated for sensitivity to hypoxia for control of ventilation [34], for metabolic rate [25] and for morphological characteristics including craniofacial shape [35]. However, specific critical windows across the entire span of embryonic development, when organogenesis, tissue differentiation and growth may be particularly vulnerable to hypoxia, have not been comprehensively investigated. Thus, our first objective was to use pulses of short-term hypoxic exposure at specific times during incubation to provide insights about when hypoxia affects key anatomical and hematological features of avian development. We hypothesized that the first third of avian incubation would prove to be the most sensitive to hypoxic exposure, based on experiments on hypoxic viability in chicken embryos cited above.

The use of the embryo of the chicken, *Gallus gallus*, as the model for studies of hypoxic effects on development has led to a considerable assemblage of data for this species. Whether the response of the chicken embryo is, in fact, actually broadly representative of all developing birds is unknown–though this is generally assumed in the absence of data to the contrary. Yet, experiments on, for example, cardiovascular development in birds suggest that there are both profound quantitative and qualitative differences in chickens compared with emus [36,37], and even between different strains of the domestic chicken [38–44]. Thus, a second purpose of this study was to generate data on hypoxic incubation effects on another bird species, the quail *Coturnix cot*urnix, and then compare these data to other bird species to begin to understand which aspects of development already documented for the chicken embryo are indeed more broadly representative of galliform birds (turkey, chicken, grouse, quail, pheasants). In this regard, we hypothesized that hypoxic exposure in quail would have similar effects to that in chickens.

Although there are many potential changes that could be induced by hypoxic exposure (see list above), this study initially documents changes in hatchability and general body features (e.g. mass, beak length, toe length) to determine general effects of hypoxia occurring at specific times in development of the quail. The study then focuses on mass measurements of the heart as well as hematological variables, to determine whether tissues/organs involved in the delivery of oxygen in the developing embryo, are most directly affected by hypoxic incubation. These data are then compared with previously published data on the domestic chicken.

## Materials and methods

### Animals and incubation

All experiments were performed with the approval of the Institutional Animal Care and Use Committee of the University of North Texas.

Eggs of the common quail (*Coturnix coturnix*) were obtained from Texas A&M University and from Bear Bayou Quail Farm, Gainesville, Texas. Upon receipt, eggs were incubated in 1.7-liter Lyon egg incubators maintained at 37°C. Each container was automatically rotated 90° every hour. Each container received one of two distinct gas mixtures produced with a Cameron gas mixer (model GF-3): either a normoxic (control) mixture with a PO_2_ of 151 mmHg (~21% O_2_) or a hypoxic PO_2_ of 110 mmHg (~15% O_2_). Oxygen levels inside the incubators were highly stable (± 2 mmHg), as confirmed daily by sampling gas from within each of the incubators, and measuring PO_2_ of that sample with a Radiometer BMS3 gas analyzer. There is no “industry standard” level of hypoxia, so 15% was chosen as being among the most commonly used level of hypoxic stress–see for example [6,25,34,45,46].

The level of relative humidity was selected based on previous protocols for hypoxic incubation of chicken eggs [2,3,25], though some studies have suggested drier air in the range of 60–70% for various species of quail [47–50]. Nonetheless, hatching rate in controls in the present study (~55%) were only slightly lower than in drier incubation conditions (63%) [48]. Humidity sampled daily in the incubators was slightly more variable than PO_2_, ranging from 75–85% RH, with an average of 79±4%RH.

### Experimental protocol

**Fig 1:**
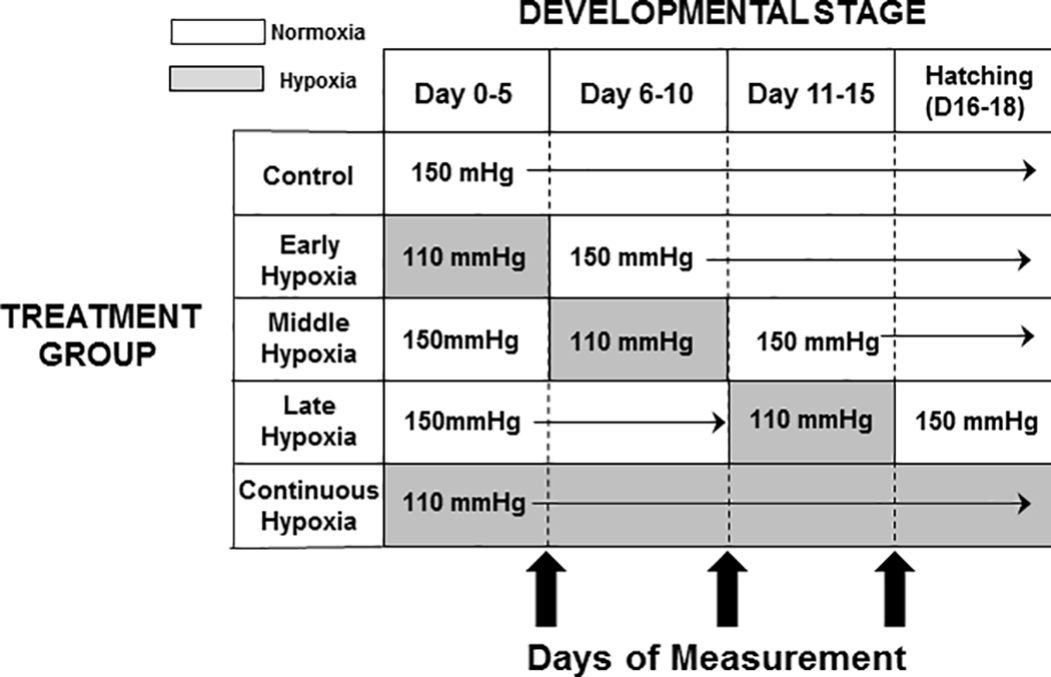
Experimental protocol for acute and chronic hypoxic treatments during the incubation of quail embryos. The shaded regions indicate hypoxic exposure.

Each shipment of incubated eggs was divided into five different groups of eggs, which were then subjected to staggered experimental protocols for hypoxic incubation. The influence of hypoxia on quail hematology has not previously been investigated at any point in development.While the focus in the chicken, for example, has often been on the last few days of development, recent studies have revealed that even pre-incubation storage conditions can alter phenotype in later embryonic development [2,51]. Consequently, the incubation period was divided into three five day periods covering all of embryonic development, based on protocols of previous studies [6,25,34]. These differentgroups were incubated in either normoxia (~150 mmHg) or different patterns and durations of hypoxia (110 mmHg), as indicated in Fig 1. The five groups were:

○ Normoxia (the control—PO_2_ of 151 mmHg throughout incubation),
○     early hypoxia (PO_2_ of 110 mmHg from D0 to D5 with normoxia for the remainder of incubation),
○     middle hypoxia (PO_2_ of 110 mmHg exposure from D6 to D10 with normoxia for all other periods of incubation),
○     late hypoxia (PO_2_ of 110 mmHg exposure from D11 to D15 with normoxia for all other periods of incubation), and
○ continuous hypoxia (PO_2_ of 110 mmHg O_2_ throughout incubation–i.e. until D16-18).

Anatomical and hematological measurements (described below) were measured in all groups at D10, 15, and at hatching (typically D16-D18).

### Viability rate

Viability for each group at each period during development was calculated as the percentage of embryos alive at D5, D10, D15 and hatching.

### Anatomical measurements

Eggs from each experimental group were assessed on D10 and D15, and at hatching. Prior to any dissection, embryos within their eggs, as well as new hatchlings, were euthanized by placing them for >10 min. in a 1 liter container with 5% Halothane vapor (Halocarbon Laboratories). Any embryos that did not appear to be alive until immediately prior to being euthanized were not analyzed. D10 and D15 embryos were removed from the egg and freed of yolk and extraembryonic membranes. Wet mass of embryos and of hatchlings was measured with a top pan balance (Denver Instrument Co.). The heart was then removed from the embryo, blotted dry, and weighed. Dry mass of embryo, hatchlings and of the heart were determined after drying for 4 days at 60°C.

Toe and beak lengths are commonly used as indices of development in the chick embryo [6,52–54]. Calipers were used to measure length of beak from tip to the anterior end of the left nostril. The length of the third toe was measured from the phalangeal-tarsometatarsal joint to the tip of the claw.

### Hematological measurements

To sample blood on D10 and 15, eggs from each group were opened at their pointed end and 1.5 ml of blood was drawn directly from a major chorioallantoic membrane artery into a heparinized glass syringe. Hatchlings were deeply anesthetized with Halothane (Halocarbon Laboratories) and blood then drawn by direct cardiac puncture.

A blood sub-sample was immediately transferred into capillary tubes, which were then sealed and centrifuged (Clay Adams—Readicrit) for 3 min at 3000 rpm to determine hematocrit (Hct). Hemoglobin concentration ([Hb]) was determined on another subsample of 30 μl of blood injected into a Radiometer OSM2 Hemoximeter. Duplicate measurements were made and averaged for both hemoglobin and hematocrit.

### Statistical analysis

Assessment for normality of distributions (Tukey-Kramer and Shapiro-Wilk normality tests) and equality of variances was followed by within- and between-group testing for statistical significance with parametric ANOVAs and Student's t-test (SPSS software). Holmes-Sidak viability data were assessed with a Kaplan-Meier logrank test (SigmaPlot software). All statistical decisions were made with a 0.05 level of significance, and are provided in Fig 1. Statistics for most within- and between-group comparisons of anatomical and hematological variables are indicated in the tables for each measured parameter. Means ± 1 Standard Error of the Mean (SEM) are provided unless otherwise indicated.

## Results

A total of 154 eggs were used in these experiments, with individual n values for each incubation condition provided in Table 1.

**Table 1:** Body wet and dry mass and water content changes during normoxic incubation and for each hypoxic incubation treatment group, measured on Day 10, Day 15, and at hatching. Means ± 1 SEM are presented. NS = not significant.

Incubation Group	Day 10				Day 15				Hatch				P for Age Effect (Horizontal Comparison—Wet Body Mass; Dry Body Mass)
	n	Body Wet Mass (g)	Body Dry Mass (g)	Body Water Content (%)	n	Body Wet Mass (g)	Body Dry Mass (g)	Body Water Content (%)	n	Body Wet Mass (g)	Body Dry Mass (g)	Body Water Content (%)	
**Normoxia (Control)**	33	2.02 ± 0.13	0.19 ± 0.03	90.7	37	6.53 ± 0.1	1.26 ± 0.04	80.7	18	8.76 ± 0.21	1.59 ± 0.04	81.9	0.001; 0.001
**Early Hypoxia**	27	1.70 ± 0.14	0.15 ± 0.03	90.9	38	5.71 ± 0.13	1.06 ± 0.03	81.4	18	8.04 ± 0.24	1.44 ± 0.05	82.1	0.001; 0.001
**Middle Hypoxia**	19	1.96 ± 0.2	0.19 ± 0.04	90.2	28	5.54 ± 0.15	0.99 ± 0.03	82.1	7	8.81 ± 0.48	1.93±± 0.31	86.2	0.001; 0.001
**Late Hypoxia**	28	1.64 ± 0.14	0.15 ± 0.03	90.7	33	5.44 ± 0.14	0.94 ± 0.03	82.7	12	8.70 ± 0.42	1.61 ± 0.05	81.5	0.001; 0.001
**Continuous Hypoxia**	47	1.50 ± 0.13	0.13 ± 0.03	91.3	10	3.40 ± 0.27	0.60 ± 0.06	80.1	-	-	-	-	0.001; 0.001
**P for Group Effect (Vertical Comparison)**		NS	NS	NS		0.001	0.001	NS		NS	NS	NS	

### Viability to hatching

**Fig 2:**
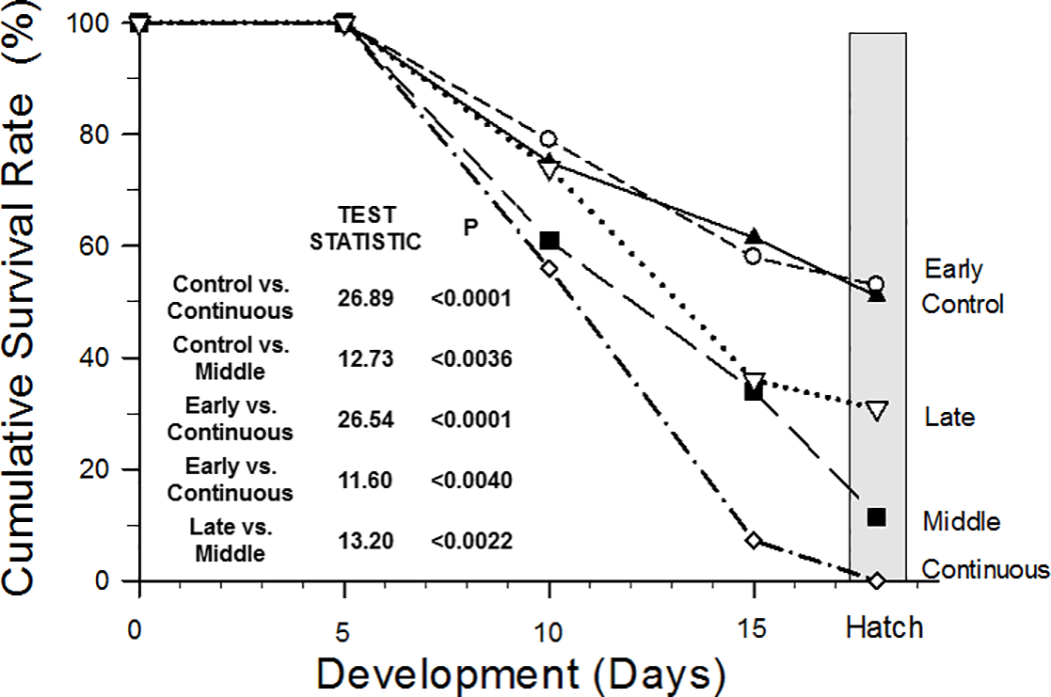
Cumulative viability rates of normoxic (control) and treatment groups of quail embryos measured at Days 10, 15, and Hatch. Table 1 provides n-values of normoxic and each treatment group. Insert contains specific comparisons, test statistics and significance values. Comparisons not shown in the inset were not significant (P>0.05)

All embryos survived all treatments through D5. Viability of the normoxic (control) group of quail embryos was ~75% and ~60% on D10 and D15, respectively, with approximately 55% of the originally incubated eggs successfully hatching (Fig 2). The early hypoxic exposure group had statistically identical viability to the control group at hatching (56%). A poorer viability at hatching was evident in the middle (~10%) and late (30%) hypoxic exposure groups. Lowest viability of all was for the continuous hypoxic group, with values of just 55%, 7% and <3% at D10, D15 and hatching, respectively.

Specific statistical comparisons of viability data are provided in the inset of Fig 2. Essentially, the middle and continuous groups showed overall statistically lower viability than the control, early and late groups.

### General observations on development

Morphologically, quail embryos in the early, middle, and late hypoxic treatment groups appeared identical to the normoxia group on all three measurement days. However, the survivors of the continuous hypoxia treatment group displayed many abnormal physiological and anatomical characteristics. At both D10 and 15, the overall appearance of the embryo was edematous, especially in the cephalic region. In addition to this profound ascites, there were also notable beak and eye deformations in the continuous hypoxic group. Deformities included incomplete or absent lower or upper beak or eye. Eyes were open by D10 in some cases, as in the other groups, but remained closed at D15 in some of the embryos from continuous hypoxia. The full length of the wing was attached to the embryonic body in some continuous hypoxia embryos, and feather development was delayed up until D15 in the continuous hypoxic group. The third toe was attached to the adjacent toes in some cases.

### Body mass

#### Normoxia

**Fig 3:**
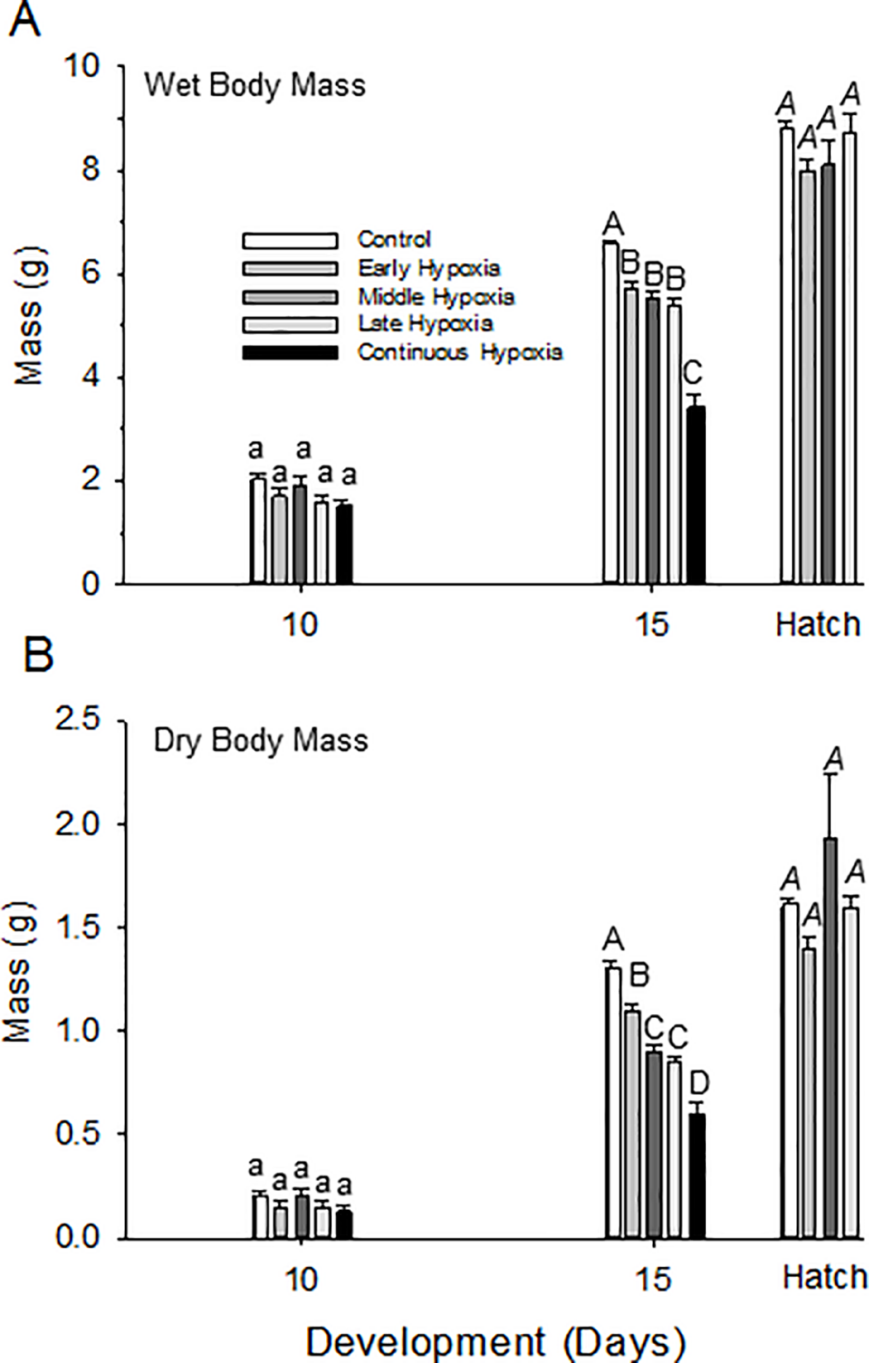
Wet body mass (A) and dry body mass (B) changes during incubation in quail embryos measured on D10, D15, and hatch for control and early, middle, late and continuous hypoxia groups. Mean ± 1 standard error is plotted. n values for each population are provided in Table 1. Statistical differences between each incubation group mean within a developmental time are indicated by letters (separate lower case, upper case or upper case italic for each developmental day).

Mean wet and dry body mass of control embryos increased significantly as a function of development (ANOVA, P<0.001), as anticipated (Table 1, Fig 3). On D10 wet and dry body mass was ~ 2.0 g and 0.2 g, respectively. Calculated water content for D10 was 90% (Table 1). By D15, there was a ~3-fold increase in wet body mass, and a ~6-fold increase in dry body mass, with water content dropping to 80%. At hatching, there was a 4-fold increase in wet body mass from D10, and 8-fold increase in dry body mass from D10 (Table 1).

#### Hypoxia

On Day 10 wet body mass of all groups that experienced hypoxia at some point during their development (early, middle, late and continuous) were not statistically different from each other(Fig 3). However, although all the wet body mass of all hypoxic groups had increased significantly on D15 compared to D10, all of the hypoxic-exposed groups showed a significantly lower wet body mass when compared to the normoxic (control) group. Notably, the early, middle and late hypoxia incubation groups increased body mass more slowly from D10 than the normoxic controls, while the body mass of the continuous hypoxia group (which died soon after D15), increased only slightly above the body mass measured 5 days previously.

Despite having a lower body mass at D15, quail embryos exposed to early, middle or late hypoxia exhibited “catch-up” growth such that there were no significant differences between these groups and the control group at hatching.

The relationships described above for wet mass generally also held for dry mass (Table 1, Fig 3B), Total water content, calculated from the mean values, was ~90% on D10, falling to ~80 on D15 and at hatch, with no discernable differences between incubation groups (Table 1).

### Beak and toe length

#### Normoxia

**Fig 4:**
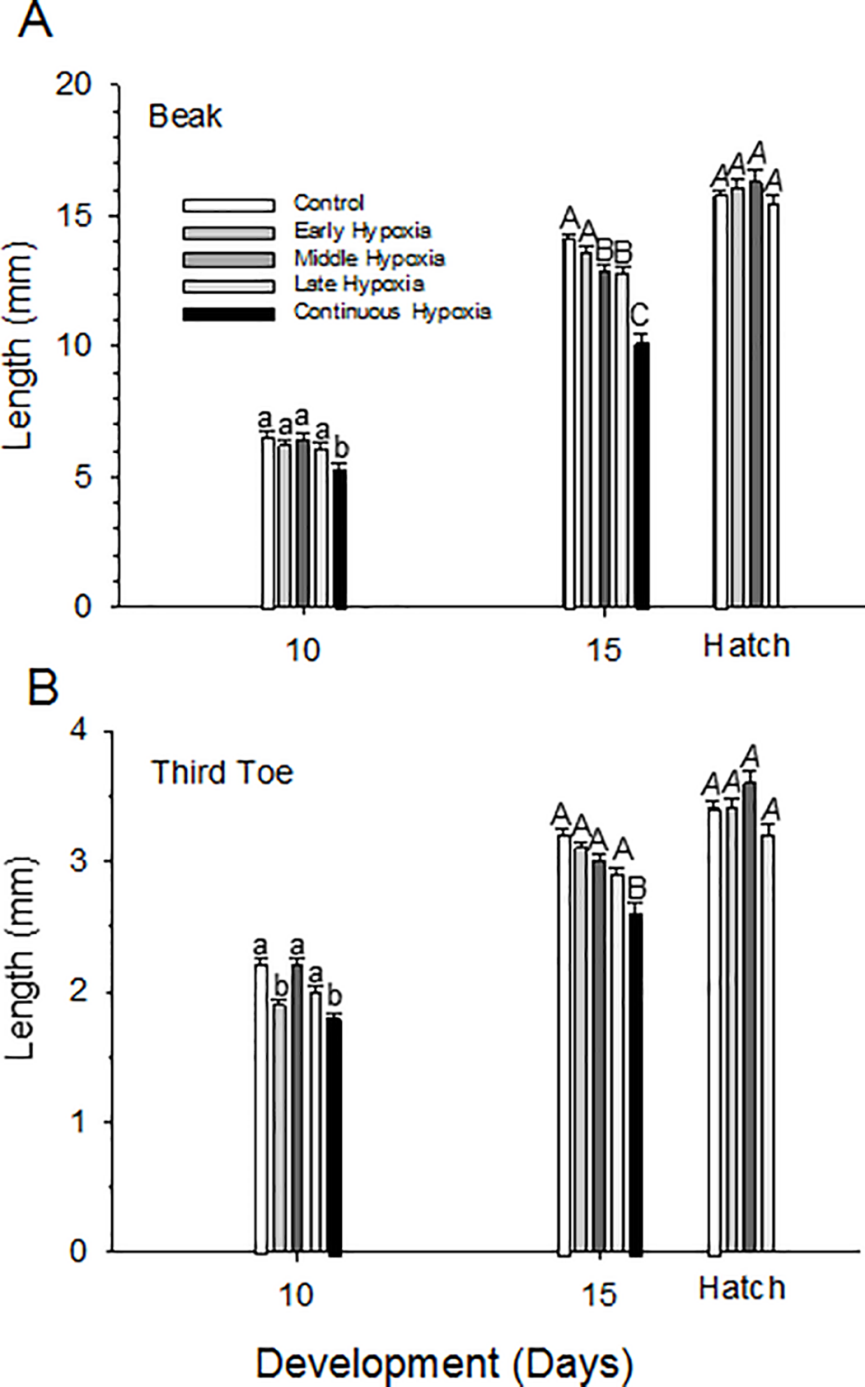
Changes in A) Beak length and (B) Third toe length during incubation in quail embryos measured on D10, D15, and hatch for control and early, middle, late and continuous hypoxia groups. Statistical differences between each incubation group mean within a developmental time are indicated by letters (separate lower case, upper case or upper case italic for each developmental day). Mean ± 1 standard error is plotted. n values provided in Table 2.

At D10, beak length averaged 2.2 ± 0.1 mm and toe lengths averaged 6.5 ± 0.2 mm (Table 2, Fig 4). By D15 beak length increased to 3.2 mm, while toe length increased to 14.1 mm (Fig 4). The beak had apparently reached maximum length by D15, and there was no significant increase in beak length (p = 0.6114) at hatching in the control population. Toe length, however, continued to increase to 15.7 mm until hatching.

**Table 2:** Toe and beak length changes during normoxic incubation and for each hypoxic incubation treatment group, measured on D10, 15, and hatching. Means ± 1 SEM are presented. NS = not significant.

Incubation Group	Day 10			Day 15			Hatch			P for Age Effect (Horizontal Comparison–Toe Length; Beak Length)
	`	Toe Length (mm)	Beak Length (mm)	n	Toe Length (mm)	Beak Length (mm)	n	Toe Length (mm)	Beak Length (mm)	
**Normoxia (Control)**	33	6.5± 0.2	2.2 ± 0.1	37	14.1 ± 0.2	3.2 ± 0.1	18	15.7 ± 0.3	3.4 ± 0.1	0.001; 0.001
**Early Hypoxia**	27	6.2± 0.2	1.9 ± 0.0	38	13.6 ± 0.2	3.1 ± 0.0	18	16.1 ± 0.3	3.4 ± 0.1	0.001; 0.001
**Middle Hypoxia**	19	6.4± 0.3	2.2 ± 0.1	28	12.9 ± 0.2	3.0 ± 0.1	7	16.3 ± 0.5	3.6 ± 0.1	0.001; 0.001
**Late Hypoxia**	28	6.1± 0.2	2.0 ± 0.0	33	12.9 ± 0.2	2.9 ± 0.1	12	15.4 ± 0.4	3.2 ± 0.1	0.001; 0.001
**Continuous Hypoxia**	47	5.3± 0.2	1.8 ± 0.0	10	10.1 ± 0.4	2.6 ± 0.1	0	-	-	0.001; 0.001
**P for Group Effect (Vertical Comparison)**		0.05	0.01		**0.01**	**0.05**		NS	NS	

#### Hypoxia

Similar absolute sizes and rates of growth in toe and beak length occurred in the early, middle and late hypoxic groups on D10 (Fig 4, Table 2). However, both beak and toe lengths were slightly but significantly lower in the late hypoxia than other populations, but this difference in both structures had disappeared by hatching. Starting with D10, the continuous hypoxia embryos showed increasingly marked reductions in both toe and beak length, before ultimately succumbing to the cumulative effects of hypoxia before hatching (Fig 4, Table 2).

### Heart mass

#### Normoxia

**Fig 5:**
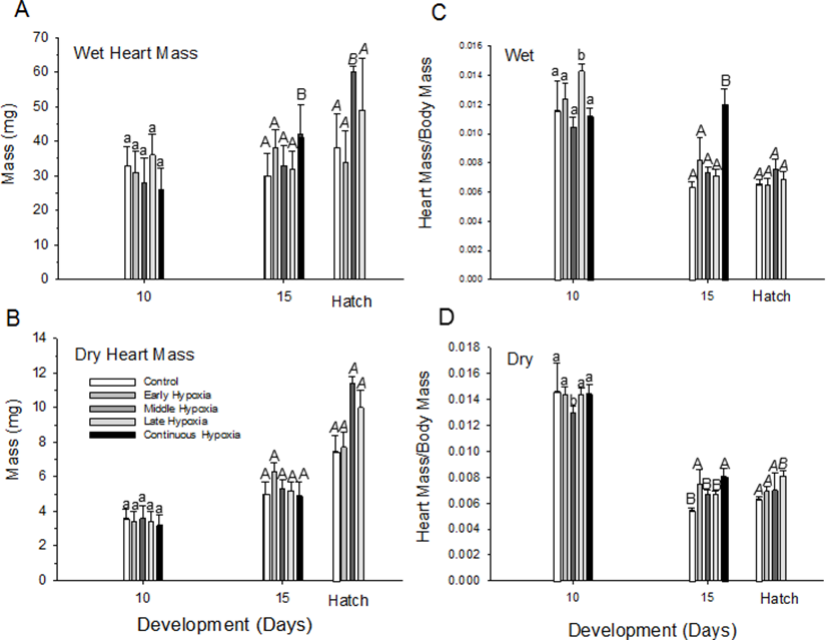
Heart mass (A, B) and heart mass to body mass ratio (C, D) changes during incubation in quail embryos for control and early, middle, late and continuous hypoxia. (Mean ± 1 standard error is plotted. n values provided in Table 3. Statistical differences between each incubation group mean within a developmental time are indicated by letters (separate lower case, upper case or upper case italic for each developmental day).

At D10, the average wet heart mass of normoxic embryos was about 33 mg, while dry heart mass was ~3.6 mg, yielding a heart water content of 89% (Table 3). The dry heart mass to dry body mass ratio was 0.015 (Fig 5). By D15, wet heart mass of normoxic embryos actually decreased slightly to 30 mg, while dry heart mass and water content remained unchanged (Fig 5) (Table 3). Dry heart mass to dry body mass ratio in the normoxic group decreased sharply to 0.006 at D15. No significant changes in wet heart mass, dry heart mass, water content or the ratio of dry heart mass to dry body mass occurred in normoxic embryos during the last few days of development before hatching (Table 3, Fig 5).

**Table 3:** Heart mass and water content changes during normoxic incubation and for each hypoxic incubation treatment group, measured on D10, 15, and hatching. Means ± 1 SEM are presented. NS = not significant.

	Day 10				Day 15				Hatch				P for Age Effect (Horizontal Comparison–Heart Wet Mass, Heart Dry Mass)
	n	Heart Wet Mass(mg)	Heart Dry Mass (mg)	Heart Water Content (%)	n	Heart Wet Mass(mg)	Heart Dry Mass (mg)	Heart Water Content (%)	n	Heart Wet Mass (mg)	Heart Dry Mass (mg)	Heart Water Content (%)	
**Normoxia (Control)**	33	33.0± 5.4^1^	3.6± 0.5	89.1	37	30.4± 6.6	5.0 ±0.7	83	18	38.5±17.3	7.5±0.9	80	0.05, 0.05
**Early Hypoxia**	27	30.9± 6	3.4±0.6	89	38	38.8±5.2	6.3 ±0.5	83	18	34.9±10.9	7.7±0.9	77	0.05, 0.05
**Middle Hypoxia**	19	28.0± 7.3	3.2 ±0.7	87	28	**33.2±5.7**	5.3 ±0.5	84	**7**	***63*.*9***± ***17*.*0***	***11*.*4***±***0*.*4***	90	0.05, 0.05
**Late Hypoxia**	28	36.7± 6	3.4±0.6	91	33	32.3±5.2	5.2 ±0.5	84	12	***49*.*4***±***15*.*4***	***12*.*8***±***1*.*0***	79	0.05, 0.05
**Continuous Hypoxia**	47	26. ±6.2	3.2±0.6	88	10	42.5±8.7	4.9 ± 0.8	88	0	-	-	-	0.05, NS
**P for Group Effect** (Vertical Comparison)		NS	NS			0.05	NS			0.05	NS		

^1^ For vertical comparisons between incubation groups at any given day, asterisks indicate values significantly different from normoxic at any given developmental stage (vertical comparisons). For horizontal comparisons within the table, values in **bold** are significantly different from the same group on D10, while values in *italics* are significantly different than both D10 and D15.

#### Hypoxia

Wet and dry heart mass increased slightly between D10 and D15 and then to a greater extent at hatching in all hypoxic incubation groups, as a consequence of overall developmental progression (Fig 5, Table 3). In all but the continuous hypoxia group, the ratio of wet heart mass to wet body mass decreased sharply from D10 to D15, leveling off and showing no further change from D15 to hatching (Fig 5). In the continuous hypoxic group, however, the ratio of wet heart mass to wet body mass remained essentially unchanged from D10 to D15, in sharp contrast to the decrease in the other groups. At the same time, the ratio of dry heart mass to dry body mass decreased significantly (P<0.0001) as in the other groups.

### Hematology

**Fig 6:**
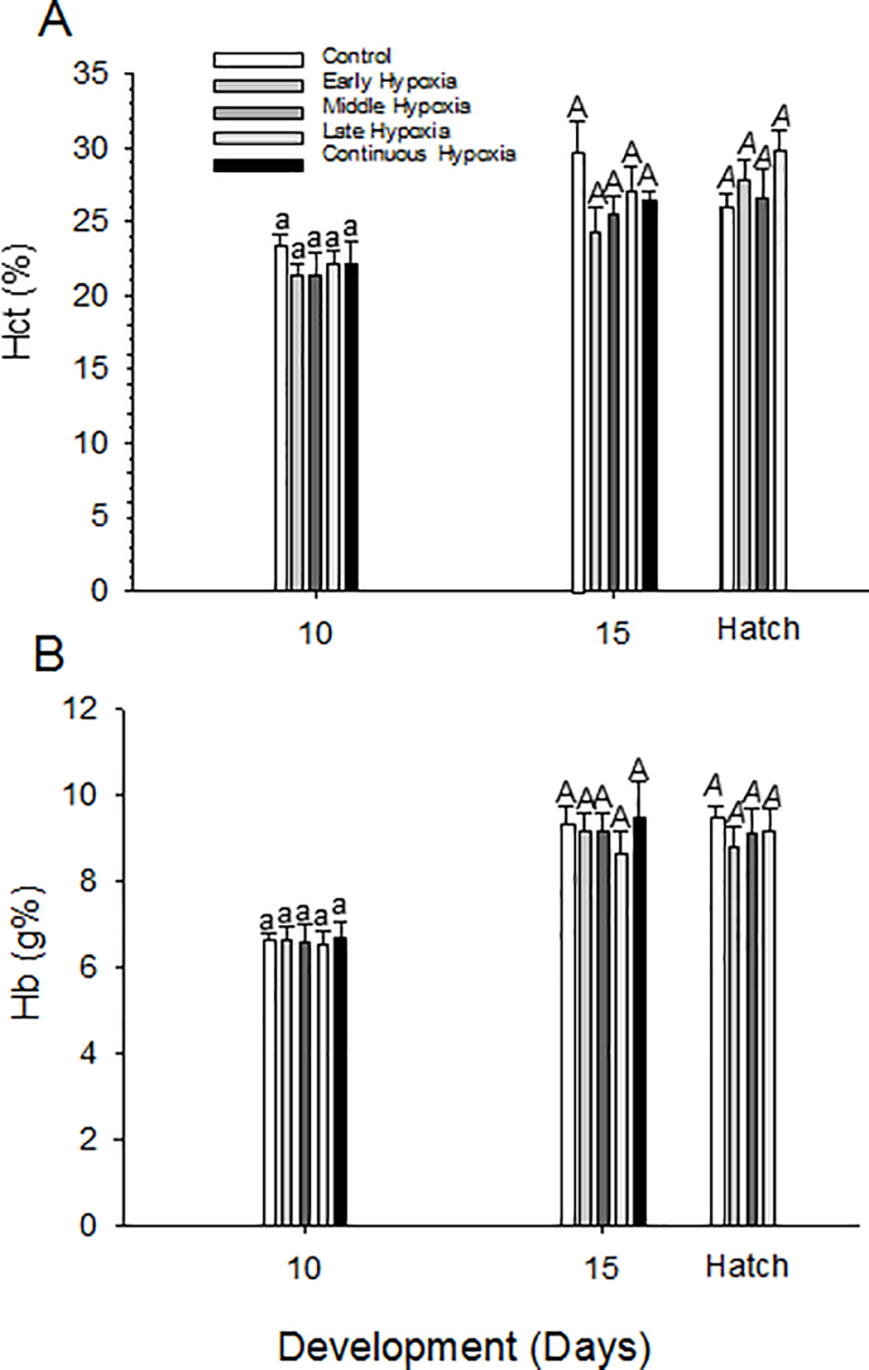
Developmental changes in (A) hematocrit and (B) hemoglobin concentration during normoxic and hypoxic incubation in quail embryos. Mean ± 1 standard error is plotted. Mean ± 1 SEM is plotted. n values for each stage are the same as provided for body mass in Table 1. Statistical differences between each incubation group mean within a developmental time are indicated by letters (separate lower case, upper case or upper case italic for each developmental day).

Hematocrit in all groups of quail embryos was 22–23% at D10, increasing to 25–29% at hatching (Fig 6B). Hemoglobin concentration was ~6.5g% at D10, increasing to ~9% at D15 and hatching (Fig 6). There were no significant differences (p>0.05) in hematocrit or hemoglobin values between the normoxic group and any hypoxic incubation group at any of the three measured points in development.

## Discussion

### Normoxic embryonic growth and viability

Growth during normoxic incubation was relatively modest through the first half of incubation, thereafter increasing more rapidly, with more than 3/4 of body mass accumulation occurring in the last 50% of incubation. This aligns with the embryos of other galliform birds [54–60], including other studies on quail [61]. Despite, or perhaps because of, these rapid morphological and physiological modifications in support of growth and development, at least in the chicken embryo there are two periods of significant low viability during normal incubation—the first week and the third week of incubation [3,55,62]. Early (<10 days incubation) non-viability in chicken embryos seems to result from irregularities in organogenesis, which is usually completed in the first week, at least in the chicken. On the other hand late (>16 days incubation) lack of viability appears due to “positioning difficulties” prior to hatching [55]. In contrast to chicken embryos, our study on quail embryos during normoxic development indicated high viability during the first third of incubation, with lac of viability beginning after day 5 of incubation. In fact, a relatively constant rate of mortality occurred throughout each measured period in incubation (Fig 2). These findings for the quail suggest no particular period of poorer viability after the first third of incubation.

### Hypoxia effects and critical windows for development

Embryonic mortality in quail embryos was greatly influenced by patterns of hypoxic exposure during incubation. Early (D0-D5) hypoxic exposure had little effect on survivability in any treatment group, and eggs hatched at approximately the same rate as the normoxic controls (Fig 2). More influential on viability was late hypoxic incubation (D11-15), which reduced hatchability from ~75% down to ~35%. Still more lethal was exposure during the middle (D6-10) of incubation (~10% viability to hatch), which was only exceeded in low viability by continuous hypoxic exposure throughout incubation (<2%). Of the various points in development in which a bout of hypoxia was experienced, exposure during middle incubation (D6-D10) proved ultimately most lethal, indicating that the most critical period for hypoxic-induced lack of viability is mid-incubation.

Not only was lack of viability progressive and highest in the continuous hypoxic group (Fig 2), but numerous sub-lethal hypoxic effects were observed throughout development: retardation of embryonic growth, edema, and deformities in beak and eye formation. Perturbations of normal growth depend not only upon when in incubation hypoxia occurs, but also with the level of hypoxia. There is an apparent dose-response effect to hypoxia in chicken embryos, with lower and/or longer oxygen levels creating more disruption to growth and size [58]. Interestingly, declines in oxygen consumption caused by mild hypoxia (15% O_2_) affect weight at hatching but otherwise have little morphological effect in chicken embryos, whereas the aerobic energy shortfall associated with severe hypoxia (10% O_2_) actually affects embryonic viability [45].

Hypoxic incubation in chicken embryos typically indicates, as for the present study on quail, that continuous hypoxia, even relatively mild, is among the most disruptive of hypoxic exposure protocols [6,25,58]. Lesser periods of brief exposure have different effects on viability depending upon when they occur. For example, several studies in chicken embryos indicate that, with respect to viability, the most hypoxia-sensitive period is early in incubation [18,20,21,26,63]. In contrast to chicken embryos, the current study on the common quail reveals that the most critical period for hypoxic exposure affecting embryo viability in the quail is the middle period of incubation. Hypoxic exposure from D6-D10 produces a progressive decrease in viability in late incubation even after restoration of full normoxia. Yet, in the common quail neither early nor late hypoxic exposure groups–both exposed to the same length and strength of hypoxia as the middle hypoxic group–experienced enhanced mortality, despite clearly showing morphological effects at the end of their hypoxic exposure period.

In the case of early hypoxic exposure, the early growth restriction induced by hypoxia can be reversed by the restoration of normoxia in incubation, so-called “catch-up growth” [26,64]. In the present study on quail, even though all hypoxic incubation induced restrictions in body mass by day 15 (Fig 3), upon hatching body mass has been restored to values not significantly different from the normoxic controls. In the case of late hypoxic exposure, quail embryos experiencing hypoxia apparently are mature enough to thwart the life-affecting influence of reduced oxygen availability. Avian embryo growth typically slows as hatching approaches, and for the last few days of incubation the embryo's weight remains relatively constant [4,52,55]. Oxygen consumption (and heart rate) similarly begin to level off in late development [1,11,65]. Thus, the ability to weather the effects of hypoxic exposure in late incubation may relate to naturally slowing demands for growth and already completed organogenesis.

It is important to note that despite early incubation being the most sensitive in chicken embryos regarding viability (see above), the metabolic responses to early, middle and late hypoxic exposure occurs in the chicken embryo appear to align more closely with the viability data for quail from the present study. Thus, hypoxic exposure in the middle of incubation in chickens leads to the most profound and lasting adjustments to oxygen consumption [25]. Apparently, long term metabolic effects resulting from hypoxic exposure can be repaired if sufficient time remains before hatching, or if the doses are sufficiently intermittent [25,45].

### Tissue-specific changes during hypoxic development

Reflecting the greatly reduced ultimate hatching success, continuous hypoxic exposure had the most debilitating effect on beak and toe development, with inhibited growth already evident by D10 and even more pronounced by D15, after which low viability occurred (Fig 4). While not directly assessed experimentally, the logical conclusion is that skeletal growth was inhibited by hypoxia, as has been shown in chicken embryos exposed to hypoxia [35,66]. The growth effects in quail appear to be a compounding of lesser effects induced earlier in incubation, since on D15 neither early nor middle groups showed any inhibition of beak or toe growth. Hypoxic exposure during late hypoxia alone was effective in diminishing beak and toe growth, suggesting a greater period of hypoxic vulnerability for these particular morphological features in late incubation, even when overall hatching success was not affected. These data in quail contrast somewhat with those for the chicken, which themselves are varied. For example, beak length was affected by early hypoxic exposure (15% O_2_) when measured in middle of incubation in chicken embryos, but additional incubation in normoxia enabled catch-up growth [67]. However, in a more recent study on chicken embryos, beak and toe length were not affected by hypoxic incubation at 13% or 15% O_2_ [58].

Heart mass (wet or dry) in quail, unlike beak and toe length, was unaffected by any combination of hypoxic exposure, including continuous exposure, an example of “organ sparing” also evident for the hearts of chicken embryos exposed to hypoxia or malnutrition [6,26]. It has been long appreciated that the effect of altered oxygen availability on the growth of individual organs in avian embryos is non-uniform [6,53,68,69], so the fact that heart mass was not affected in the quail embryo, while beak and toe length growth was inhibited, is not unheralded. However, we had anticipated cardiac hypertrophy, since the heart would be a key participant in any hypoxia-induced increase in blood flow to tissues during incubation. Heart mass (especially ventricular mass) generally correlates with stroke volume in vertebrates [70]. Although changes in heart morphology that would indicate changes in heart performance didnot occur, this does not mean that stroke volume and cardiac output do not actually change. Certainly, cardiovascular responses to hypoxia have been well documented in the middle to late embryos of galliform and ratite birds [36–38] [71–74] and may have occurred in the present study on quail embryos.

Interestingly, the ratio of wet heart mass to body mass in the present study on quail showed some significant changes with hypoxia incubation. Most striking was the larger heart mass to body mass ratio evident in the continuous hypoxic group on D15 (Fig 5) compared to other populations. Since this aberration was not evident in the ratio of dry heart mass to dry body mass, it would appear that the increased ratio reflects major increases in water content of the heart or relatively dehydration of the body in the continuous hypoxic group. This finding is consistent with the general observations of fluid imbalances in continuous hypoxic embryos, none of which survived further hypoxic incubation to hatching.

### Hypoxia-induced hematological changes

Despite protocols that included intervals of 5 day hypoxic incubation as well as continuous hypoxic incubation, no significant changes in hematology were measured in any of the quail embryo groups. During early development chicken embryos are similarly unable to counter hypoxic incubation by mounting an increased production of red blood cells and/or the expansion of total blood volume [75], despite the fact that such responses are present in the last week of incubation [46,76–78]. Why would a polycythemic hypoxic response, so very typical for many adult vertebrates, be lacking in early avian embryos, especially in a development stage viewed as highly plastic? One explanation, of course, is that exposure to 15% oxygen is simply an inadequately potent stimulus. Yet, the fact that morphological pathologies were induced shows that our protocol exceeded some form of hypoxic threshold. An alternative explanation is that during at least the first two thirds of development erythropoiesis is already occurring at maximum rate [79]. Yet another explanation is that the erythropoietic system is not sufficiently mature to mount a polycythemic response to hypoxia that is typical of adult birds. Increased erythropoiesis would, of course, increase blood viscosity, which would increase apparent peripheral resistance [80]. The chronic increase in cardiac afterload that would accompany elevated blood viscosity during an early critical window could interfere with normal heart development, which in part depends on a precise balance of hemodynamic forces [70]. Thus, natural selection may have led to a muted response early in development.

Though an adaptive polycythemic response is lacking, potentially reduced oxygen transport during hypoxic incubation in quail embryos could be offset by production of hemoglobin(s) with a greater affinity for oxygen, as occurs in other birds [79,81–84]. Chicken embryos produce at least two distinct forms of hemoglobin. Hemoglobin with greater O_2_ affinity is produced earlier in development, and is replaced gradually by hemoglobin with lower affinity [15,79,85]. Blood O_2_ transport in quail embryos could also be enhanced by changes in blood O_2_ affinity due to alterations in red blood cell organophosphates such as ATP, as has been documented in chicken and turkey embryos [15,86]. Decreases in [ATP] increase blood O_2_ affinity in chicken embryos exposed to hypoxia [15,87,88]. Future investigations of embryonic quail blood should consider whether, despite unchanged numbers of red blood cells, the O_2_ carrying characteristics of quail blood were temporarily or permanently modified by hypoxic incubation.

### Quail embryo as a model for study of developmental hypoxia

The domestic chicken (*Gallus gallus*) is indisputably the main avian model for developmental studies–for recent reviews see [1,89]. The quail is a lesser utilized avian model, though it has figured prominently in studies of skeletal myogenesis [90], early heart development [91–93] and sex differentiation [94], to name just a few foci of developmental studies using quail. The assumption in these studies is that quail are similar if not identical to the chicken embryo in many if not all basic aspects of their developmental biology.

The use of model organisms (or organisms assumed to be like another model organism) has greatly advanced our understanding of development within every area of biology. However, an unduly narrow focus on models to the exclusion of exploring species diversity can also lead to a distorted perception of what is “representative” [89,95]. For example, there is considerable contrast between the domestic chicken (*Gallus gallus*) and non-domesticated wild birds with respect to the reduction of metabolism, hatchability, growth rate, and hatching mass upon exposure to moderate hypoxia related to altitude [4]. Similarly, domestic chickens show major differences from the emu (*Dromiceius novaehollandiae*) in the pattern of onset of key cardiovascular control mechanism–see [36,38,96]. Additionally, any precocial bird is likely to be a less than perfect model for development in an altricial bird, and vice versa. The present study of embryos of the quail (*Coturnix coturnix*) reports additional key differences in responses compared to chicken embryos, in viability rates during normal development, and also in the primary period of vulnerability (critical window) for growth and morphology during incubation.

### Conclusion

Continuous hypoxia is clearly not well tolerated in quail embryos, leading to high rates of mortality. Yet, embryos experiencing a mixture of hypoxia and normoxia at various times during incubation were capable of sufficient self-repair and/or catch-up growth over time so as to appear morphologically identical at hatching, even though they followed significantly different developmental trajectories in arriving at this common phenotype.

Collectively, these findings for avian embryonic development suggests that avian embryos exposed to hypoxia during incubation at various stages in development are pushed into abnormal developmental trajectories, but once hypoxic incubation is replaced by normoxic incubation, development of surviving embryos not only progresses, but follows a novel developmental trajectory leading back towards the normal hatchling phenotype for quail. Though the developmental trajectory is not a normal one, it may provide the additional time to repair earlier hypoxia-induced damage.

Future experiments should be directed at determining dose-response relationships, which will reveal potentially differential susceptibilities to hypoxia by different tissues and organ systems. Additionally, such studies may additionally reveal “soft edges” to the critical windows that are not readily revealed by a simple two dimensional approach to susceptible periods [29,97]. A more precise identification of the critical windows for physiological processes, in addition to morphological structures, will also help elucidate the overall developmental process in birds. Finally, the extent to which the developmental responses of the quail compare with the chicken and other precocial and altricial birds will reveal how much interfamily variation exists in development process in birds.

## Supporting information

S1 File Table with supporting data for Fig 2 survival. (DOCX)

S2 File Table with supporting data for Fig 3 body masses. (DOCX)

S3 File Table with supporting data for Fig 4 Toe beak length. (DOCX)

S4 File Table with supporting data for Fig 5 heart masses. (DOCX)

S5 File Table with supporting data for Fig 6 hematology. (DOCX)
