# Supplementary material for: Critical developmental windows for morphology and hematology revealed by intermittent and continuous hypoxic incubation in embryos of quail (Coturnix coturnix)
Source: PLoS One. 2017 Sep 19;12(9):e0183649. doi: 10.1371/journal.pone.0183649 (PMC5604962; doi:10.1371/journal.pone.0183649)
Supplement: S1 File — (DOCX) [file pone.0183649.s001.docx]

| Supporting Data for FIGURE 2 Survival Data | | | | | |
| --- | --- | --- | --- | --- | --- |
| Day of Incubation | Control | Early Hypoxia | Middle Hypoxia | Late Hypoxia | Continuous Hypoxia |
| 0 | 100 | 100 | 100 | 100 | 100 |
| 5 | 100 | 100 | 100 | 100 | 100 |
| 10 | 75 | 79 | 61 | 74 | 56 |
| 15 | 61.5 | 58 | 34 | 36 | 7.3 |
| Hatch | 51 | 53 | 11.5 | 30.9 | 0.07 |
