# Supplementary material for: Critical developmental windows for morphology and hematology revealed by intermittent and continuous hypoxic incubation in embryos of quail (Coturnix coturnix)
Source: PLoS One. 2017 Sep 19;12(9):e0183649. doi: 10.1371/journal.pone.0183649 (PMC5604962; doi:10.1371/journal.pone.0183649)
Supplement: S2 File — (DOCX) [file pone.0183649.s002.docx]

| Supporting Data for FIGURE 3 BodyMasses | | | | | | |
| --- | --- | --- | --- | --- | --- | --- |
| Incubation Day | Mean Control Wet Body Mass | se | Mean Early Hypoxia Wet Body Mass | se | Mean Middle Hypoxia Wet Body Mass | se |
| 10 | 2.02 | 0.13 | 1.7 | 0.14 | 1.9 | 0.2 |
| 15 | 6.53 | 0.1 | 5.7 | 0.13 | 5.5 | 0.15 |
| Hatch | 8.76 | 0.2 | 8 | 0.2 | 8.097 | 0.483 |
| Incubation Day | Mean Control Dry Body Mass | se | Mean Early Hypoxia Dry Body Mass | se | Mean Middle Hypoxia Dry Body Mass | se |
| 10 | 0.2 | 0.03 | 0.15 | 0.03 | 0.2 | 0.04 |
| 15 | 1.3 | 0.04 | 1.1 | 0.03 | 0.9 | 0.03 |
| Hatch | 1.6 | 0.04 | 1.4 | 0.05 | 1.93 | 0.31 |

| Mean Late Hypoxia Wet Body Mass | se | Mean Continuous Hypoxia Wet Body Mass | se |
| --- | --- | --- | --- |
| 1.6 | 0.14 | 1.5 | 0.13 |
| 5.4 | 0.14 | 3.4 | 0.27 |
| 8.7 | 0.4 |  |  |
| Mean Late Hypoxia Dry Body Mass | se | Mean Continuous Hypoxia Dry Body Mass | se |
| 0.15 | 0.03 | 0.13 | 0.03 |
| 0.85 | 0.03 | 0.6 | 0.06 |
| 1.6 | 0.05 |  |  |
