# Supplementary material for: Critical developmental windows for morphology and hematology revealed by intermittent and continuous hypoxic incubation in embryos of quail (Coturnix coturnix)
Source: PLoS One. 2017 Sep 19;12(9):e0183649. doi: 10.1371/journal.pone.0183649 (PMC5604962; doi:10.1371/journal.pone.0183649)
Supplement: S3 File — (DOCX) [file pone.0183649.s003.docx]

| Supporting Data for FIGURE 4 Toe and Beak | | | | | | |
| --- | --- | --- | --- | --- | --- | --- |
| Incubation Day | Mean Control Toe Length | SE | Mean Early Hypoxia Toe Length | SE | Mean Middle Hypoxia Toe Length | SE |
| 10 | 6.5 | 0.24 | 6.2 | 0.21 | 6.4 | 0.31 |
| 15 | 14.1 | 0.23 | 13.6 | 0.21 | 12.9 | 0.24 |
| 17.5 | 15.7 | 0.3 | 16.1 | 0.34 | 16.3 | 0.5 |
| Incubation Day | Mean Control Beak Length | SE | Mean Early Hypoxia Beak Length | SE | Mean Middle Hypoxia Beak Length | SE |
| 10 | 2.2 | 0.05 | 1.9 | 0.04 | 2.2 | 0.06 |
| 15 | 3.2 | 0.05 | 3.1 | 0.04 | 3 | 0.05 |
| 17.5 | 3.4 | 0.06 | 3.4 | 0.07 | 3.6 | 0.09 |

| Mean Late Hypoxia Toe Length | SE | Mean Continuous Hypoxia Toe Length | SE |
| --- | --- | --- | --- |
| 6.1 | 0.22 | 5.3 | 0.2 |
| 12.8 | 0.22 | 10.1 | 0.42 |
| 15.4 | 0.4 |  |  |
| Mean Late Hypoxia Beak Length | SE | Mean Continous Beak Length | SE |
| 2 | 0.04 | 1.8 | 0.04 |
| 2.9 | 0.05 | 2.6 | 0.09 |
| 3.2 | 0.08 |  |  |
