# Supplementary material for: Critical developmental windows for morphology and hematology revealed by intermittent and continuous hypoxic incubation in embryos of quail (Coturnix coturnix)
Source: PLoS One. 2017 Sep 19;12(9):e0183649. doi: 10.1371/journal.pone.0183649 (PMC5604962; doi:10.1371/journal.pone.0183649)
Supplement: S4 File — (DOCX) [file pone.0183649.s004.docx]

| Supporting Data for FIGURE 5 - Heart Wet and Dry Mass | | | | | | | |
| --- | --- | --- | --- | --- | --- | --- | --- |
| Incubation Day | Mean Control Wet Heart Mean | se | Mean Early Hypoxia Wet Heart Mean | se | Mean Middle Hypoxia Wet Heart Mean | se | Mean Late Hypoxia Wet Heart Mean |
| 10 | 33 | 5.4 | 31 | 6 | 28 | 7.3 | 36 |
| 15 | 30 | 6.6 | 38 | 5.2 | 33 | 5.7 | 32 |
| Hatch | 38 | 10 | 34 | 9 | 60 | 1.7 | 49 |
| Incubation Day | Mean Control Dry Heart Mean | se | Mean Early Hypoxia Dry Heart Mean | se | Mean Middle Hypoxia Dry Heart Mean | se | Mean Late Hypoxia Dry Heart Mean |
| 10 | 3.6 | 0.5 | 3.4 | 0.6 | 3.6 | 0.7 | 3.4 |
| 15 | 5 | 0.7 | 6.3 | 0.5 | 5.3 | 0.5 | 5.2 |
| Hatch | 7.5 | 0.9 | 7.7 | 0.9 | 11.4 | 0.4 | 10 |

| se | Mean Continuous Wet Heart Mean | se |
| --- | --- | --- |
| 6 | 26 | 6.2 |
| 5 | 42 | 8.7 |
| 15 | | |
| se | Mean Continuous Dry Heart Mean | se |
| 0.6 | 3.2 | 0.6 |
| 0.5 | 4.9 | 0.8 |
| 1 | | |
