# Supplementary material for: Critical developmental windows for morphology and hematology revealed by intermittent and continuous hypoxic incubation in embryos of quail (Coturnix coturnix)
Source: PLoS One. 2017 Sep 19;12(9):e0183649. doi: 10.1371/journal.pone.0183649 (PMC5604962; doi:10.1371/journal.pone.0183649)
Supplement: S5 File — (DOCX) [file pone.0183649.s005.docx]

| Supporting Data for FIGURE 6 Hematology | | | | | | |
| --- | --- | --- | --- | --- | --- | --- |
| Incubation Day | Mean Control Hct | se | Mean Early Hypoxia Hct | se | Mean Middle Hypoxia Hct | se |
| 10 | 23.406 | 0.78 | 21.381 | 0.763 | 21.375 | 1.511 |
| 15 | 29.625 | 2.154 | 24.231 | 1.784 | 25.533 | 1.146 |
| hatch | 26 | 0.92 | 27.833 | 1.409 | 26.6 | 1.913 |
| Incubation Day | Mean Control Hb | se | Mean Early Hypoxia Hb | se | Mean Middle Hypoxia Hb | se |
| 10 | 6.586 | 0.196 | 6.668 | 0.272 | 6.605 | 0.386 |
| 15 | 9.34 | 0.411 | 9.186 | 0.404 | 9.186 | 0.404 |
| hatch | 9.428 | 0.345 | 8.814 | 0.454 | 9.1 | 0.575 |

| Mean Late Hypoxia Hct | se | Mean Continuous Hypoxia Hct | se |
| --- | --- | --- | --- |
| 22.211 | 0.772 | 22.154 | 1.476 |
| 27.036 | 1.683 | 26.5 | 0.5 |
| 29.875 | 1.315 |  |  |
| Mean Late Hypoxia Hb | se | Mean Continuous Hypoxia Hb | se |
| 6.542 | 0.291 | 6.641 | 0.403 |
| 8.645 | 0.497 | 9.4 | 0.9 |
| 9.188 | 0.489 |  |  |
